# Effects of trans‐ versus cis‐resveratrol on adrenergic contractions of the rat tail artery and role of endothelium

**DOI:** 10.14814/phy2.14666

**Published:** 2020-12-24

**Authors:** Ian R. VanAntwerp, Laura E. Phelps, Jacob D. Peuler, Phillip G. Kopf

**Affiliations:** ^1^ Department of Pharmacology Midwestern University Downers Grove IL USA

**Keywords:** adrenergic contractions, cis‐isomer, endothelium, rat tail artery, resveratrol

## Abstract

The health benefits of the natural polyphenol trans‐resveratrol may play an important role in preventing a variety of diseases. Resveratrol has been shown to reduce blood pressure and improve metabolic diseases such as type 2 diabetes mellitus and obesity. Our previous studies examined the role of K^+^ channels in the vasorelaxation responses to trans‐resveratrol in the rat tail artery. During these studies, we uncovered a novel transient contraction prior to the sustained relaxation effect of trans‐resveratrol. Thus, the purpose of this study was to determine the role of the endothelium in these vascular contraction and relaxation responses to trans‐resveratrol. We additionally sought to determine if the cis‐isomer of resveratrol exerts any of the same vascular effects as the trans‐isomer. The vascular responses to trans‐resveratrol were examined in rat tail arteries with intact or denuded endothelium over a 2‐hr period. Additionally, the vascular responses to trans‐ and cis‐resveratrol were compared in rat tail arteries with intact endothelium. Both the transient contractile response and the persistent relaxation response to trans‐resveratrol were similar in the arterial rings with intact or denuded endothelium. There was a significant correlation between the initial contraction‐enhancing action of trans‐resveratrol and the magnitude of the sustained relaxation for vessels with both intact and denuded endothelium. Moreover, we demonstrated that cis‐resveratrol produced a significantly greater relaxation response as compared to trans‐resveratrol without the initial contractile response. These data demonstrate the role of the vascular smooth muscle in the vascular responses to resveratrol and the potential clinical benefits of the cis‐isomer of resveratrol as compared to the trans‐isomer.

## Graphical abstract

The goal of these studies was to determine (a) if trans‐resveratrol's ability to initially contract and then relax rat tail arterial tissue was dependent on the presence of intact endothelium and (b) if resveratrol's abilities to initially contract and then relax rat tail arterial tissue occur with the cis‐isomer as well as the trans‐isomer of resveratrol.

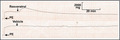

## 1 INTRODUCTION

The health benefits of trans‐resveratrol as evidenced by both preclinical experiments and clinical trials in humans suggest that this natural polyphenol, commonly known for its powerful antioxidant action, may play an important role in preventing a variety of diseases (Belguendouz et al., 1997; Bhatt et al., 2012; Brown et al., 2010; Chan et al., 2011; Dolinsky et al., 2013; Iervasi et al., 2012; Liu et al., 2005; Patel et al., 2011; Rivera et al., 2009; Timmers et al., 2011; Toklu et al., 2010). In hypertensive rat models, oral resveratrol has been shown to improve cardiovascular function by chronically lowering arterial pressure (Chan et al., 2011; Dolinsky et al., 2013; Liu et al., 2005; Rivera et al., 2009; Toklu et al., 2010) and preventing cardiac hypertrophy (Dolinsky et al., 2013; Liu et al., 2005). In human clinical trials, resveratrol has not only been shown to reduce arterial pressure chronically but also induce metabolic changes such as improving glycemic control in patients with type 2 diabetes mellitus (Bhatt et al., 2012) or obesity (Timmers et al., 2011).

Given its ability to lower arterial pressure, it is not surprising that several published studies have consistently shown that resveratrol can directly relax precontracted smooth muscle in various arteries in vitro (Gojkovic‐Bukarica et al., 2008; Naderali et al., 2000; Nagaoka et al., 2007; Novakovic, Gojkovic‐Bukarica, et al., 2006; Shen et al., 2013). One vessel not studied by others is the long ventral tail artery of the laboratory rat, despite being widely recognized as a convenient, inexpensive, and valid model for many other arteries throughout the body (Medgett, 1985; Rajanayagam & Medgett, 1987; Souza et al., 2008). Our lab has successfully used the rat tail artery as a model for studying the mechanisms of direct relaxant effects of other agents also known for lowering arterial pressure chronically (Peuler et al., 1999, 2004; Peuler & Phelps, 2015; Phelps & Peuler, 2010). Therefore, the original overall purpose of our studies was to further investigate resveratrol's arterial relaxant effect using the rat tail artery as an in vitro model.

These early studies sought to determine if trans‐resveratrol's vasorelaxant action is greater in the distal (resistance) versus proximal (conductance) portion of the rat tail artery. We found that the half‐maximally effective concentration values were essentially identical (25 ± 3 versus 27 ± 3 μM) for relaxing precontracted rings prepared from distal versus proximal tissues (Stom et al., 2016). This observation contrasted with a previous report of greater relaxation in resistance versus conductance arteries (Naderali et al., 2000). We additionally determined that these resveratrol‐induced relaxations could not be blocked by any of seven different K^+^ channel blockers (Stom et al., 2016); some of which had already been shown to do so in other isolated arteries in vitro (Gojkovic‐Bukarica et al., 2008; Nagaoka et al., 2007; Novakovic, Bukarica, et al., 2006; Novakovic, Gojkovic‐Bukarica, et al., 2006; Shen et al., 2013).

However, during these studies, we uncovered a novel unanticipated action not yet reported. In our arterial ring preparations, trans‐resveratrol transiently stimulated contraction well before its sustained relaxant effect became apparent (Stom et al., 2016). This action provided the first reasonable explanation for previously unexplained increases in arterial pressures observed during acute intravenous administration of resveratrol as a protective antioxidant to animal models of traumatic ischemic tissue injury, in which oxidative stress and hypotension are often present (Hamburger et al., 2013; Kaplan et al., 2005) and in need of correction (Toumpoulis, 2006). Also unanticipated, this transient contraction was notably inhibited by some of the same K^+^ channel blockers (particularly tetraethylammonium, TEA, and glibenclamide) that failed to influence its relaxant effect. Although we did not rule out smooth muscle as a possible site for such a paradoxical finding, we suspected that resveratrol could also be acting on K^+^‐selective mechanosensitive ion channels located in the endothelium where they may participate in the release of contracting factors (Nilius & Droogmans, 2001).

Thus, the purpose of the following experiments was to determine if the endothelium plays a role in trans‐resveratrol's ability to both initially contract and then later relax rat tail arteries that were precontracted with an adrenergic agonist. We additionally sought to determine if the cis‐isomer of resveratrol exerts any of the same vascular effects as the trans‐isomer on endothelium‐intact rings from the same vessel. Because in nature the cis‐isomer is the far less stable of the two (Gambini et al., 2015; Wang & Chatterjee, 2017), most research up to now (including all our own) has been conducted with the trans‐isomer. A sufficiently stable preparation of the cis‐isoform for research purposes was not available until recently. A new commercially available stable preparation of the cis‐isomer (Cayman Chemicals, Ann Arbor, MI) has made these comparisons of the cis‐ and trans‐isomers possible.

## 2 METHODS

### 2.1 Reagents

Krebs buffer ingredients, DMSO, phenylephrine (PE), and saponin were purchased from Sigma Aldrich (St, Louis, MO). Cis‐ and trans‐resveratrol were purchased from Cayman Chemical (Ann Arbor, MI).

### 2.2 Isolation and preparation of arterial contractile tissue rings

Adult male Sprague‐Dawley rats were sacrificed for removal of a portion of the ventral tail artery. We employed this vessel because of its widespread recognition as a convenient, inexpensive, and valid model for other arteries (Medgett, 1985; Rajanayagam & Medgett, 1987; Souza et al., 2008). As studied and reviewed by Souza *et al*. (Souza et al., 2008), while its proximal portion more resembles conductance arteries, its distal portion is more similar functionally and structurally to resistance vessels throughout the body (small arteries and arterioles). The endothelium of the distal is easier to damage while handling compared to the proximal. Thus, for our purposes, the proximal portion of each vessel that we isolated was cleaned and carefully sectioned into multiple 3‐mm cylindrical rings using a bound set of evenly spaced scalpel blades. In our experience, individual rings sectioned in this manner exhibit more uniform contractile responses than if sectioned with multiple cuts by a single blade.

### 2.3 Isometric tension recording

A maximum of six vascular rings were selected at random for experimental treatments on each particular day. Each ring was mounted between two tungsten wire stirrups and suspended in a 40‐ml tissue bath and allowed to equilibrate for several minutes before experimentation at a resting tension of 1,500 mg in standard physiological (Krebs) buffer (11‐mM D‐glucose) at 37°C and gassed to pH 7.4 with regulated delivery of O_2_/CO_2_. For experiments that required the removal of the endothelium, the vessels were perfused for 2 min with saponin (0.1 mg/ml buffer) at room temperature prior to hanging, as described previously (Graser et al., 1988), and as we have successfully employed before with rat tail arterial tissue (Phelps & Peuler, 2010). All tensions for these tissues were recorded (in mg units) with the aid of force transducers connected to an 8‐channel Grass paper chart recorder (six working channels).

To examine the relaxant effects of resveratrol, vessels were precontracted with the alpha‐1 agonist PE at its half‐maximally effective concentration of 0.5 μM. In our experience, PE produces steady, sustained contractions when compared to other contractile agonists previously used in other resveratrol studies (Naderali et al., 2000; Shen et al., 2013). After these first contractions plateaued, acetylcholine (ACh) at 10 μM was administered to investigate each ring for the presence or absence of intact endothelium. A vessel that relaxed greater than 50% was considered to have intact endothelium. In experiments where vessels were denuded of endothelium, a relaxation response of less than 5% was used as an indicator of successful denudation. The ring segments were then washed in physiological buffer two to three times prior to the following experiments.

### 2.4 Role of endothelium in vascular responses to trans‐resveratrol

To examine the role of the endothelium in the vascular response to trans‐resveratrol, half of the blood vessels were denuded and half were left with intact endothelium. Precontracted vessels were exposed to 26‐μM trans‐resveratrol or vehicle (DMSO). Trans‐resveratrol at 26 μM is the half‐maximally effective relaxing concentration from our previous work, which demonstrated a transient contraction that peaks at 6–12 min followed by sustained relaxation (Stom et al., 2016). DMSO‐treated segments acted as a control to detect any falloff of PE‐induced contraction. All vessels were observed for 2 hr following administration of trans‐resveratrol or vehicle.

### 2.5 Comparative vascular responses to cis‐ and trans‐resveratrol

The vascular effects of cis‐ and trans‐resveratrol were examined in endothelium‐intact vascular rings. Vessels were split into treatment groups and concurrently administered either 60‐μM trans‐resveratrol or 60‐μM cis‐resveratrol. 60 μM was chosen because that concentration demonstrated a significant difference between the two in terms of inhibition of calcium currents through membrane‐bound voltage‐gated calcium channels (VGCCs) in cultured arterial A7r5 cells (derived from rat embryonic aorta) (Campos‐Toimil et al., 2007). All vessels were observed for 2 hr following administration of resveratrol.

### 2.6 Data analysis

Numerical values were determined from paper chart recordings of the various tissue contractile tension parameters (as defined in the above studies). Statistical evaluation of the trans‐resveratrol experiments involved subjecting the numerical values to analysis of variance (ANOVA) followed by appropriate mean comparison tests (Bonferroni's) for detection of statistically significant effects of the different experimental conditions. For the experiments examining cis‐ and trans‐resveratrol, unpaired *t* tests were considered the most appropriate to achieve the same end. Data were presented in the form of mean ± *SEM* with a *p*‐value for significance equal to or less than .05.

## 3 RESULTS

### 3.1 Role of endothelium in vascular responses to trans‐resveratrol

**Figure 1:**
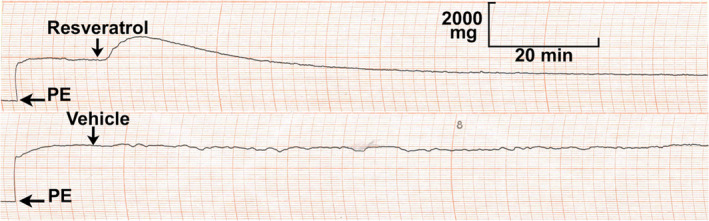
Representative chart recordings demonstrating the effects of 26‐µM trans‐resveratrol (resveratrol) and its vehicle on phenylephrine (PE)‐induced contractions of vascular rings from the proximal portion of the rat tail artery with endothelium removal achieved with saponin. Shown is a transient increase in contraction immediately after administration of resveratrol followed by delayed sustained decrease (above). Lack of such effects are shown after administration of vehicle (below). Similar effects were observed among vascular rings with endothelium‐intact. Each PE = 0.5 µM

Representative chart recordings demonstrate the effects of trans‐resveratrol and its vehicle (DMSO) on PE‐induced contractions to vascular rings with endothelium‐removed (Figure 1). Immediately following the administration of trans‐resveratrol, the vessels contract, followed by a sustained relaxation over 2 hr. In vehicle control, there is a lack of a vascular effect and the PE precontraction remains stable over the 2‐hr period. We observed similar responses to trans‐resveratrol and vehicle among rings with intact endothelium.

**Figure 2:**
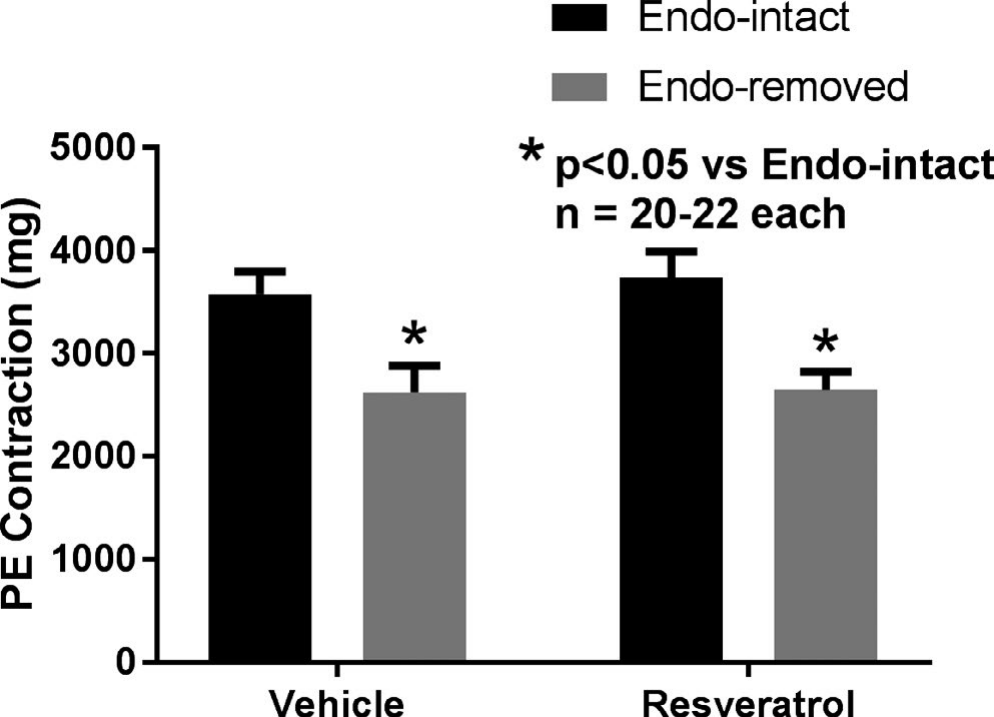
Effect of endothelium removal on the magnitude of the second PE‐induced contractions observed prior to addition of trans‐resveratrol (resveratrol) or its vehicle. *p < .05 versus Endo‐intact represents a statistically significant difference from two‐factor ANOVA not only when vehicle and resveratrol data are combined, but also when analyzed separately

Endothelial denudation significantly reduced the magnitude of PE precontractions prior to the addition of vehicle or trans‐resveratrol (endothelium‐intact: 3,734 ± 255 mg, endothelium‐removed: 2,651 ± 173 mg, *p* < .05) (Figure 2). However, there was no significant difference in the magnitude of PE precontractions between vessels that received resveratrol or vehicle.

**Figure 3:**
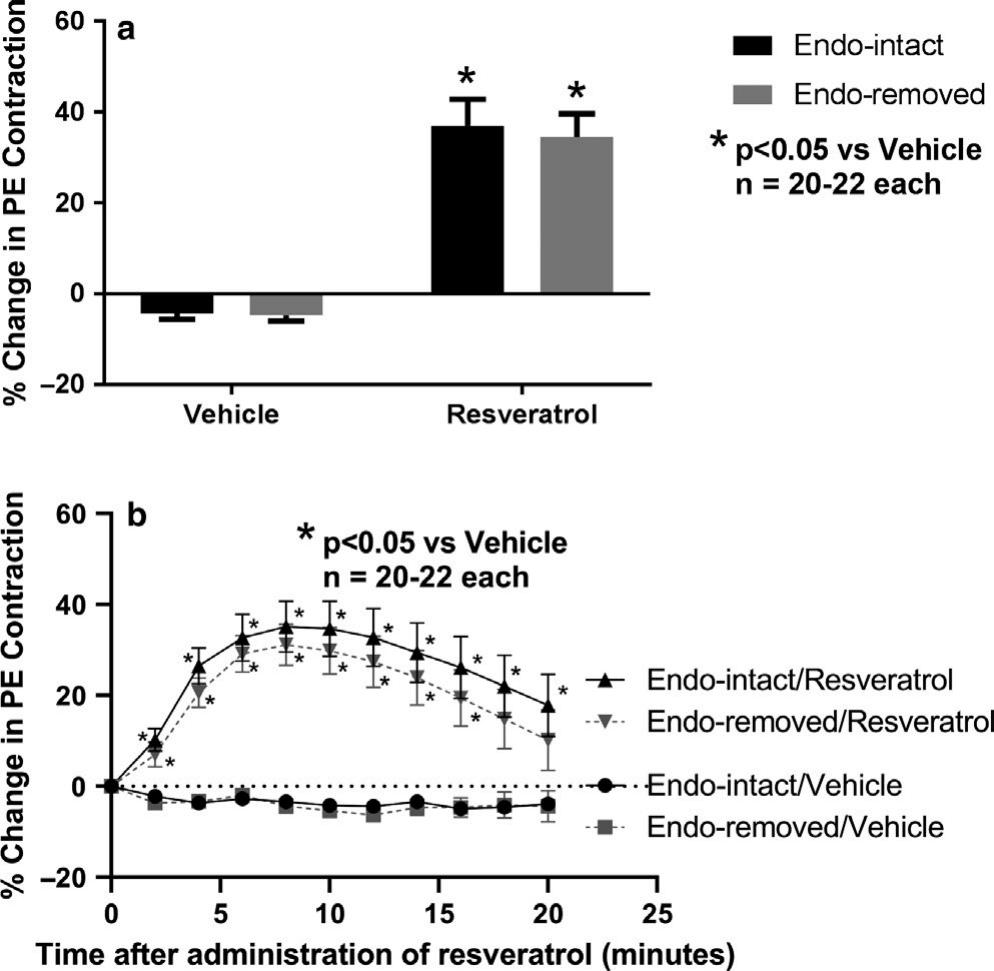
Effect of endothelium removal on initial (8–9 min) maximal changes in second PE‐induced contractions after addition of trans‐resveratrol (resveratrol) or its vehicle (a) and the time course of such changes as observed from 2 to 20 min (b). Calculated as percent change in magnitude of PE‐induced contractions observed immediately before addition of resveratrol or its vehicle to the magnitude observed later at the various time points. For a, the duration of 8–9 min was calculated from the mean time to peak increases after addition of resveratrol and applied then to the vehicle group in order to calculate its related percent changes. *p < .05 versus vehicle represents a statistically significant difference from two‐factor ANOVA for comparisons between resveratrol and vehicle in both endothelium‐intact and endothelium‐removed rings but not for removal of the endothelium itself. All vascular rings administered resveratrol demonstrated transient increases in PE‐induced contractions

Trans‐resveratrol induced transient contractions which peaked at 8–9 min (Figure 3). This finding was observed in all rings that were administered trans‐resveratrol including both endothelium‐intact and endothelium‐removed rings. Such contractions were not observed in any rings that were given the vehicle DMSO. These peak trans‐resveratrol‐induced contractions were significantly different from their respective time points in vehicle controls (endothelium‐intact: vehicle, −4.4 ± 1.2%; trans‐resveratrol, 37.0 ± 5.8%; *n* = 20–22, *p* < .05) (endothelium‐removed: vehicle, −4.7 ± 1.2%; trans‐resveratrol, 34.6 ± 5.0; *n* = 20–22, *p* < .05) (Figure 3a). Removal of the endothelium had no effect on this initial contraction response. When plotted at 2‐min intervals, trans‐resveratrol induced statistically significant contractions between 2 and 20 min as compared to vehicle control in the endothelium‐intact vessels and between 2 and 16 min as compared to vehicle control in the endothelium‐removed vessels (Figure 3b). Removal of the endothelium had no effect on this contraction response over the 20‐min period.

**Figure 4:**
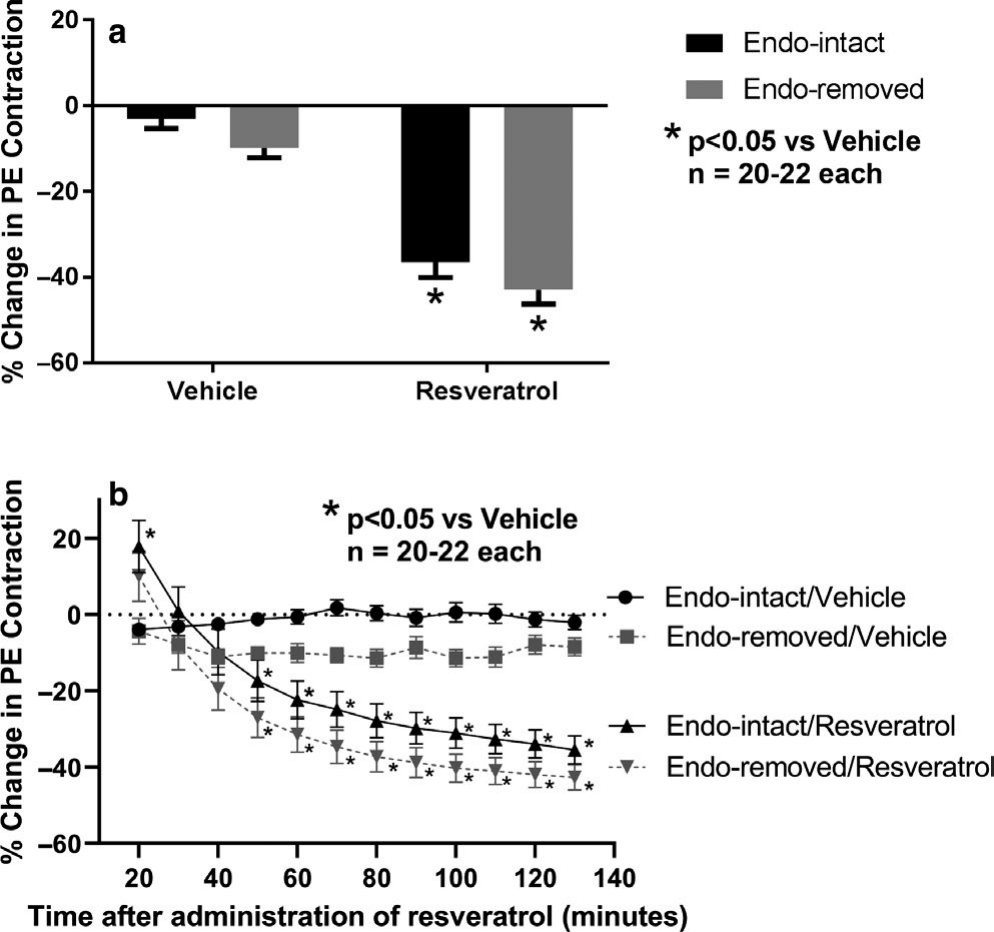
Effect of endothelium removal on final (2 hr) changes in second PE‐induced contractions observed after the addition of trans‐resveratrol (resveratrol) or its vehicle (a) and the time course of such changes as observed from 20 to 130 min (b). Calculated as percent change in magnitude of contractions observed immediately before addition of resveratrol or its vehicle to the magnitude observed later at the various time points. At all times from 40 to 130 min, two‐factor ANOVA revealed an overall statistically significant main factor effect of endothelium removal (*p* < .05) for both resveratrol‐ and vehicle‐treated rings, but only when analyzed in combination not separately by Bonferroni's mean comparisons *p < .05 versus vehicle represents a statistically significant effect from two‐factor ANOVA for comparisons of resveratrol versus vehicle in both endothelium‐intact and endothelium‐removed rings

After this transient contraction phase over the first 20 min, trans‐resveratrol induced statistically significant relaxations which plateaued after 2 hr (endothelium‐intact: vehicle, −3.0 ± 2.3%; trans‐resveratrol, −36.5 ± 3.6%; *n* = 20–22, *p* < .05) (endothelium‐removed: vehicle, −9.8 ± 2.3%; trans‐resveratrol, −43.0 ± 3.3%; *n* = 20–22, *p* < .05) (Figure 4a). There was no statistically significant difference indicated for endothelium‐intact versus endothelium‐removed vessels when compared within treatment groups. However, when results from trans‐resveratrol and vehicle treatments were analyzed in combination, there was a statistically significant effect of endothelium removal. When plotted at 10‐min intervals, trans‐resveratrol induced statistically significant relaxations between 50 and 130 min as compared to vehicle control in both endothelium‐intact and endothelium‐removed vessels (Figure 4b).

**Figure 5:**
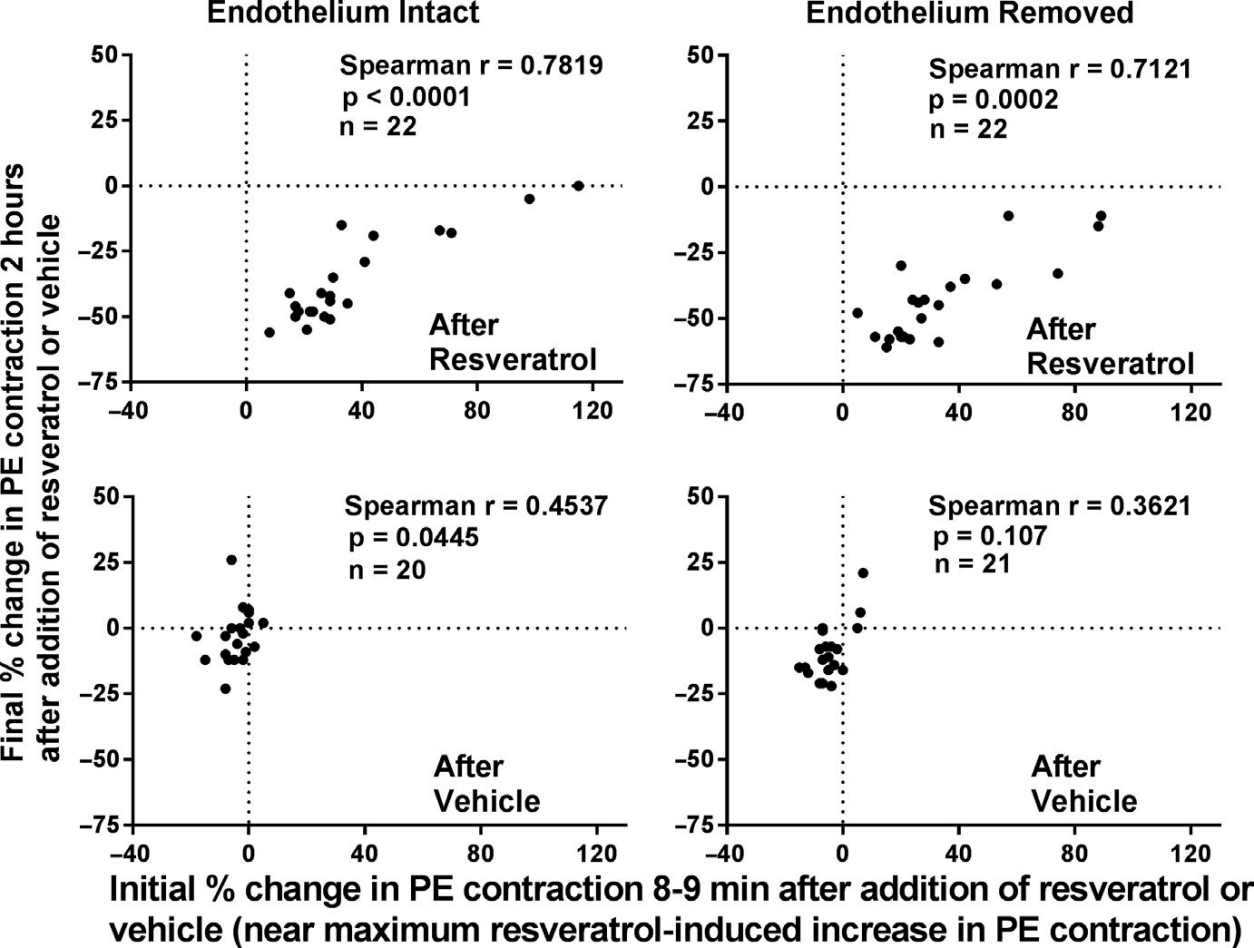
Spearman's correlation coefficients (*r*) relating final (2 hr) changes to initial (8–9 min) changes in PE‐induced contractions following addition of trans‐resveratrol (resveratrol) or its vehicle to arterial rings with endothelium‐intact or endothelium‐removed. Spearman's correlation coefficients were statistically significant after addition of resveratrol to both endothelium‐intact and endothelium‐removed arterial rings but smaller in magnitude after addition of vehicle to all rings and then only significant for endothelium‐intact rings

In order to determine if the initial trans‐resveratrol‐induced contractions are having an effect on the degree of relaxation that is produced by trans‐resveratrol at 2 hr, Spearman's correlation coefficients (*r* values) were calculated (Figure 5). For rings administered trans‐resveratrol, Spearman's *r* values were statistically significant (endothelium‐intact: 0.7819, *p* < .0001; endothelium‐removed: 0.7121, *p* < .0002). Thus, there is a statistically significant correlation between the initial contraction response of trans‐resveratrol and the magnitude of the relaxation response of trans‐resveratrol at the end of 2 hr in both endothelium‐intact and endothelium‐removed rings alike. Most importantly, the correlation indicates that a greater initial contraction response results in a less substantial relaxation response. For rings administered vehicle, Spearman's *r* values were variable and the magnitude of the r values was notably smaller for vehicle versus trans‐resveratrol‐treated rings overall (endothelium‐intact: 0.4537, *p* = .0445; endothelium‐removed: 0.3621, *p* = .107).

### 3.2 Comparative vascular responses to cis‐ and trans‐resveratrol

**Figure 6:**
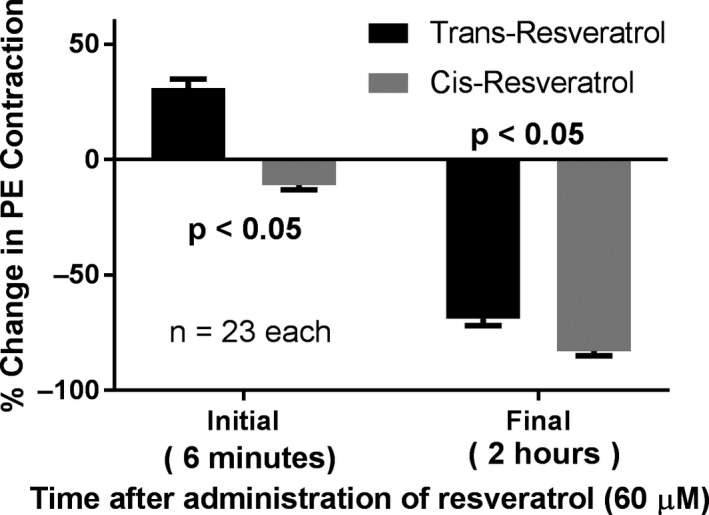
Initial (6 min) and final (2 hr) changes in PE‐induced contractions after addition of trans‐ and cis‐resveratrol to rat tail arterial rings; calculated as percent change in the magnitude of the PE‐induced contractions observed immediately before such additions. The duration of 6 min was the average time to the initial peak increases after addition of the trans‐resveratrol and was therefore applied then to the cis‐resveratrol group in order to calculate its related percent changes. *p* < .05 indicates statistically significant differences between the trans‐ versus cis‐resveratrol, at both the 6‐min and the 2‐hr times

Another set of vascular experiments were sought to determine if the cis‐isomer of resveratrol had the same capacity to initially contract and then relax rat tail arterial tissue as that of the trans‐isomer of resveratrol (Figure 6). The expected transient contraction response to trans‐resveratrol began immediately and reached a maximum magnitude (peak values), an average of 6 min after its addition. This enhancement was observed in all rings that were given the trans‐resveratrol, but was not observed in any of the rings that were given cis‐resveratrol (trans‐resveratrol: 31 ± 4%, cis‐resveratrol: −11 ± 2%, *p* < .05). However, the maximal relaxation response to trans‐resveratrol after 2 hr was significantly less than cis‐resveratrol (trans‐resveratrol: −69 ± 3%, cis‐resveratrol: −83 ± 2%, *p* < .05).

## 4 DISCUSSION

The goal of these studies was to determine (a) if trans‐resveratrol's ability to initially contract and then relax rat tail arterial tissue was dependent on the presence of intact endothelium and (b) if these same vascular responses to trans‐resveratrol in rat tail arterial tissue occur with the cis‐isomer of resveratrol as well.

In our previous work, we were not able to identify the precise mechanism whereby trans‐resveratrol initially enhanced adrenergic contractions in our tail artery tissues (Stom et al., 2016). We suspected that the endothelium was involved because vascular endothelial cells possess mechanosensitive ion channels which when stretched can directly alter the release of endothelial contracting factors or indirectly alter the ability of various endogenous agonists to release contracting factors (Harder, 1987; Hishikawa & Lüscher, 1997; Katusic et al., 1987; Nilius & Droogmans, 2001; Nilius et al., 1997; Sekiguchi et al., 1996; Vanhoutte, 1987; Yang et al., 2002). In our previous work, we found that the K^+^ channel blocking agents TEA and glibenclamide notably inhibited the magnitude of the initial transient contractions caused by trans‐resveratrol (Stom et al., 2016). Some mechanosensitive ion channels in the endothelium are known to be K^+^‐selective (Nilius & Droogmans, 2001) and therefore are potential sites for a K^+^ channel‐related action of trans‐resveratrol. Additionally, some of the endothelium‐dependent contractions, which have been observed in response to excess stretch of the wall of some arteries, have been reported as transient in duration (Katusic et al., 1987; Vanhoutte, 1987) and thus similar in that respect to what we observed with trans‐resveratrol (Stom et al., 2016). However, in the present work, we observed that the initial transient contractions caused by trans‐resveratrol were not endothelium‐dependent.

Thus, in the future, we will reexamine the role of the vascular smooth muscle in these initial transient contractions. Glibenclamide has been reported to inhibit contractions produced by prostaglandin F2 alpha in rings prepared from rat aorta and canine femoral, mesenteric, renal, coronary, and cerebral arteries (Zhang et al., 1991). The authors concluded that glibenclamide was acting specifically at the level of the arterial smooth muscle receptor for only prostaglandin F2 alpha and/or its intracellular smooth muscle signal transduction pathway, but not on glibenclamide‐sensitive K^+^ channels within the smooth muscle cell membrane (Zhang et al., 1991). We will conduct future studies to determine if glibenclamide (and perhaps TEA as well) is acting similarly to inhibit the contraction‐enhancing action of trans‐resveratrol in the rat tail artery, possibly at a yet to be identified polyphenol receptor site on smooth muscle that specifically binds trans‐resveratrol.

Another goal of these studies was to determine the role of the endothelium in trans‐resveratrol's ability to relax the rat tail artery. In our previous work, K^+^ channel blockers did not alter trans‐resveratrol's relaxant effect in our rat tail artery segments (Stom et al., 2016). Thus, we thought that the opening of smooth muscle K^+^ channels was an unlikely mechanism responsible for trans‐resveratrol's relaxing action in the rat tail artery. Based on this data, we hypothesized that trans‐resveratrol's ability to relax PE‐induced contractions of rat tail artery might be endothelium‐dependent. Endothelial relaxing functions are controlled through the release of various vasorelaxant factors, including nitric oxide (NO), which are responsible for mediating vascular relaxation of the underlying smooth muscle (Lerman & Zeiher, 2005; Luksha et al., 2009; Woodman et al., 2000). Resveratrol has been shown to increase nitric oxide synthase (NOS) expression in human umbilical vein endothelial cells (Wallerath et al., 2002) and has been shown to induce NO production in the renal artery of normal rats by an endothelial‐dependent pathway (Gordish & Beierwaltes, 2014). Resveratrol has also been extensively studied for its powerful antioxidant properties (Boydens et al., 2015; Burns et al., 2002; Dolinsky et al., 2013; Gliemann et al., 2016; Gordish & Beierwaltes, 2014; Huang et al., 2001; Kiziltepe et al., 2004; Lancon et al., 2016; Toklu et al., 2010; Xia et al., 2017), which may oppose the ability of reactive oxygen species to rapidly inactivate NO (Kalinowski et al., 2002). Resveratrol improved endothelial dysfunction in spontaneously hypertensive rats in vivo (Bhatt et al., 2011) and its relaxing action was significantly diminished following chemical removal of endothelium in PE precontracted rat corpus cavernosum in vitro (Dalaklioglu & Ozbey, 2013). The vasorelaxant effect of resveratrol was blocked by l‐NAME, a NOS inhibitor, in isolated rat thoracic aorta segments precontracted with PE in vitro (Chen & Pace‐Asciak, 1996). Removal of the endothelium reduced resveratrol's relaxant effect in isolated porcine retinal arterioles and blockade of NOS mimicked this effect (Nagaoka et al., 2007).

However, our present results demonstrate that trans‐resveratrol still relaxed our denuded tail artery segments. Other isolated arterial tissue studies examining endothelial‐independent mechanisms have targeted smooth muscle K^+^ channels, with some reporting significant effects of K^+^ channel blockers (Gojkovic‐Bukarica et al., 2008; Nagaoka et al., 2007; Novakovic, Bukarica, et al., 2006; Novakovic, Gojkovic‐Bukarica, et al., 2006; Shen et al., 2013). Consideration for smooth muscle calcium channels has received less attention but could be a focus of our future studies. Resveratrol was able to almost completely relax endothelium‐denuded mesenteric rat artery precontracted with K^+^ and this effect was completely abolished with nifedipine, a calcium channel blocker (Gojkovic‐Bukarica et al., 2008). This would support a theory that inhibition of arterial smooth muscle VGCCs may play a role in resveratrol's relaxing action through a K^+^ channel‐independent mechanism (Gojkovic‐Bukarica et al., 2008), consistent with our previous work in which trans‐resveratrol's relaxing action was unaltered by K^+^ channel blockers in our tail arterial tissues (Stom et al., 2016). Thus, VGCC could be a potential focus of our future studies.

Additionally, the relaxation response to trans‐resveratrol appeared to be greater in denuded tail artery segments. Our vehicle‐treated denuded ring segments also showed a decreased level of PE contraction when compared to vehicle‐treated rings with intact endothelium over the 2‐hr experiment. Thus, this greater effect is potentially due to some nonspecific action of the saponin‐based endothelium removal method we employed on all our denuded arterial ring segments. For example, saponin is known to sometimes weaken smooth muscle by altering its cell and microsomal membranes (Kwan et al., 1988; Su & Zhang, 1989).

Furthermore, it is possible that the mechanism responsible for trans‐resveratrol's initial transient contraction of the rat tail artery persists and limits its ability to produce sustained relaxations at the end of 2 hr. As our results showed, there was a statistically significant correlation between the initial contraction effect of trans‐resveratrol (at 8–9 min) and the magnitude of the sustained relaxation (at the end of 2 hr) for both endothelium‐intact and endothelium‐removed rings alike. Thus, the mechanism responsible for trans‐resveratrol's vascular contraction effect may not be transient, but rather persist over the entire duration in which the sustained relaxations are observed. Future studies aiming to identify and understand the precise mechanisms for both the initial contraction response and sustained relaxing action of trans‐resveratrol should consider this important correlation. This observation may suggest that the contractile effect may limit the ability of the relaxation effect to contribute to long‐term lowering of blood pressure in chronically hypertensive patients.

The second goal of this study was to determine if resveratrol's vascular contraction and relaxation effects in rat tail arterial tissue occur with the cis‐isomer as well as the trans‐isomer of resveratrol. Although cis‐resveratrol (just as trans‐resveratrol) has already been shown by others to inhibit VGCC in cultured arterial smooth muscle cells (Campos‐Toimil et al., 2007), a mechanism which could potentially explain its relaxation of arterial smooth muscle, we are the first to actually demonstrate such relaxation in freshly isolated intact arterial tissue. More importantly, we found that relaxation to be greater than that produced by trans‐resveratrol. However, the cis‐isomer inhibits VGCCs to a lesser degree than the trans‐isomer (Campos‐Toimil et al., 2007). Thus, while inhibition of such channels may explain the relaxant effect of both, it cannot explain why relaxation by the cis form was greater.

**Figure 7:**
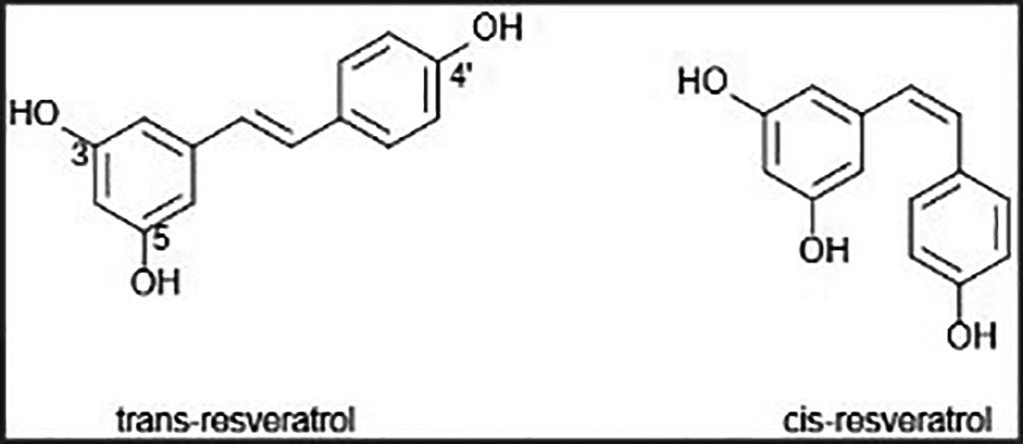
The comparative chemical structures of trans‐ and cis‐resveratrol

In addition, our data would suggest a potential reason why the relaxation response is greater with the cis‐isomer is that it lacks the mechanism responsible for the initial contraction response, which may persist with the trans‐isomer. Thus, in the suggested future study of searching for a ligand‐receptor interaction at a possible polyphenol receptor site on arterial smooth muscle, the cis‐isomer could become an important control substance in uncovering such a receptor to then possibly explain trans‐resveratrol's contraction‐enhancing action. We speculate that the isomeric bend in cis‐resveratrol is preventing a ligand‐receptor interaction of a hydroxyl group either due to shape or steric hindrance (Figure 7). For example, the hydroxyl group at carbon 3 remains fully open for interaction in both the trans‐ and cis‐isomers, whereas the hydroxyls at carbons 5 and 4 are more crowded in the cis‐isomer. Also, resveratrol is a stilbene derivative. The cis‐isomer of stilbene is sterically hindered and forces the aromatic rings out of plane. The more nonplanar property of the cis form of stilbene may explain why it is less stable than the trans‐isomer, which may be true for cis‐ versus trans‐resveratrol as well (Wang & Chatterjee, 2017).

One major clinical implication of this finding is that, by exerting a greater arterial relaxant effect than the trans‐isomer, cis‐resveratrol could potentially exert a greater arterial pressure‐lowering effect in hypertensive patients. There is a need for a more effective lowering of blood pressure by resveratrol in such patients as other reviewers have already discussed (Hamza & Dyck, 2014; Liu et al., 2015). Unfortunately, except for the one expensive preparation of cis‐resveratrol itself, available now only for research purposes (Cayman Chemicals, Ann Arbor, MI), all other natural sources are much too unstable for practical daily use. Thus, what is needed is synthesis of new derivatives of the cis‐isomer that are more chemically stable for routine clinical use and yet retain the beneficially greater arterial relaxant effect that we uncovered in the present study. Up to now, others have commonly focused on synthesizing derivatives of the trans‐isomer, especially to increase its potency for the prevention and/or treatment of cardiovascular diseases (Ruan et al., 2012). Our results now clearly suggest re‐focusing on derivatives of the cis‐isomer. Furthermore, our results clearly implicate the rat tail artery as an excellent model vessel with which to identify them.

## CONFLICTS OF INTEREST

The authors declare that they have no conflict of interest.

## AUTHOR CONTRIBUTIONS

Ian R. VanAntwerp: Collected the data, contributed data or analysis tools, and performed the analysis. Laura E. Phelps: Collected the data and contributed data or analysis tools. Jacob D. Peuler: Conceived and designed the analysis, contributed data or analysis tools, performed the analysis, and wrote the paper. Phillip G. Kopf: Conceived and designed the analysis, contributed data or analysis tools, and wrote the paper.
